# When Overdose of Doxylamine Leads to Severe Rhabdomyolysis and Renal Failure That Requires Hemodialysis: A Case Report and Literature Review

**DOI:** 10.7759/cureus.43395

**Published:** 2023-08-12

**Authors:** Bair Cadet, Salman Bhutta, Samaan Mahmoudzadeh, Marie Merisier, Nickul Shah

**Affiliations:** 1 Nephrology, Nassau University Medical Center, East Meadow, USA; 2 Nephrology, Long Island Jewish Medical Center, Queens, USA; 3 Medicine, American University of Caribean School of Medicine, Cupecoy, SXM; 4 Quality, Woodhull Medical Center, Brooklyn, USA; 5 Medicine, Broad Institute, Cambridge, USA

**Keywords:** nephrotoxicity, doxylamine, paresthesia, non-traumatic rhabdomyolysis, drug-induced acute renal failure

## Abstract

A 52-year-old male with acute onset right-sided weakness, numbness, and buttock pain after consuming 30 tablets of doxylamine antihistamine the night prior. Laboratory tests showed elevated creatinine kinase, blood urea nitrogen, creatinine, troponins, liver transaminases, and phosphate. The patient was admitted to the medical intensive care unit for severe rhabdomyolysis, acute liver failure, and acute kidney injury secondary to doxylamine intoxication. Studies describe symptoms of severe doxylamine intoxication, such as impaired consciousness (coma), grand mal seizures, and cardiopulmonary arrest. Circulating myoglobin causes oxidative injury to the kidney through the formation of F2-isoprostanes leading to renal vasoconstriction. One study explained drug-induced rhabdomyolysis via two mechanisms: direct drug injury to the striated muscle and local muscle compression in seizure, coma, and metabolic abnormality. Treatment involves aggressive hydration with monitoring of serum electrolytes and renal function. Aggressive volume expansion via intravenous fluids remains critical in preventing rhabdomyolysis-associated nephrotoxicity and myoglobin-induced acute renal failure. Alkalinization of urine may prevent renal vasoconstriction resulting in enhanced excretion of the toxic metabolites of doxylamine and myoglobin via renal tubules, thereby reducing peak serum concentration time and preventing direct renal tissue damage.

## Introduction

Rhabdomyolysis describes the damage and breakdown of skeletal muscle, causing myoglobin, intracellular proteins, and electrolytes to leak into circulation, leading to potentially life-threatening complications [1]. The United States reports 26,000 cases of rhabdomyolysis annually [1,2]. Though rhabdomyolysis occurs in patients with agitation or seizure events, it also manifests in those who overdose on doxylamine. Doxylamine is a first-generation H1 antihistamine possessing hypnotic, anticholinergic, and local anesthetic effects, with a half-life of roughly 10 hours and metabolization via the liver [3,4]. Doxylamine overdose increased in recent years due to its availability as an over-the-counter drug commonly used as a nighttime sleep aid. Clinical studies describe symptoms of severe doxylamine intoxication, such as impaired consciousness (coma), grand mal seizures, and cardiopulmonary arrest [5, 6]. Although the pathophysiologic mechanism remains unclear, clinicians report several factors which may contribute to the development of rhabdomyolysis or acute renal failure-related (ARF) secondary to rhabdomyolysis in non-traumatic injury [2, 7, 8].

Timely recognition and prompt treatment of rhabdomyolysis in patients typically results in complete recovery, effectively minimizing the risk of serious complications. Clinical management for rhabdomyolysis involves intensive hydration while closely monitoring serum electrolyte levels and renal function. In cases where seizures occur secondary to doxylamine intoxication and anticholinergic effects, diazepam is used as the initial treatment, followed by phenytoin and phenobarbital [9]. Despite interventions and treatments, it is essential to note that rhabdomyolysis resulting from drug abuse can result in acute renal failure, which may necessitate immediate initiation of dialysis therapy [10].

This article was previously presented as a meeting abstract at the 2022 American Society of Nephrology meeting on November 05, 2022

## Case presentation

A 52-year-old male with a past medical history of hypertension (HTN) and opioid dependence presented to the emergency department (ED) for acute onset right-sided weakness, numbness, and right buttock pain. The patient reported taking 30 doxylamine tablets the night before arrival in ED due to difficulty sleeping. That night, the patient experienced nausea and one non-bloody, non-bilious vomiting episode. Upon awakening the next day, six hours before arrival in the ED, the patient stated he experienced right lower extremity (RLE) numbness/tingling. He attempted to stand but could not bear weight and fell, striking his right upper extremity (RUE). The patient returned to bed and went back to sleep. However, upon awakening, the patient endorsed persistent weakness of RUE and RLE with numbness of RLE, prompting their visit to the ED. The patient reports a history of use of pain medications for chronic back pain. He returned from detox two weeks prior and was prescribed a seven-day supply of Suboxone with a plan to follow up for detox. However, the patient could not afford a return trip and subsequently completed their prescription for Suboxone. The patient states that he took multiple doxylamine tablets to fall asleep and recover from withdrawal symptoms. Upon admission, the patient presented with elevated creatinine kinase (CK) >100,000, blood urea nitrogen (BUN)/ creatinine (Cr) 71/5.8, liver transaminases (LFTs) aspartate aminotransferase (AST) 2170, alanine aminotransferase (ALT) 536, and phosphate 7.9. The patient had positive urine toxicology for opiates and doxylamine.

The patient was initially worked up for an aortic dissection, provided neurologic and laboratory findings. He was then admitted to the medical intensive care unit (MICU) for severe rhabdomyolysis, acute liver failure, and acute kidney injury (AKI) secondary to doxylamine intoxication. His LFTs, CK, BUN/Cr, and cardiac markers were trended throughout their admission. The patient received n-acetylcysteine for acute liver failure and was started on aggressive intravenous (IV) hydration. He remained oliguric, and an indwelling catheter was placed on admission to the MICU. The patient received a left femoral Shiley catheter for emergent hemodialysis on hospital day one, and the patient was started on hemodialysis on day two. Throughout their hospital stay, the patient received three cycles of hemodialysis with the removal of 330mL once and 2.5L twice. 

**Figure 1:**
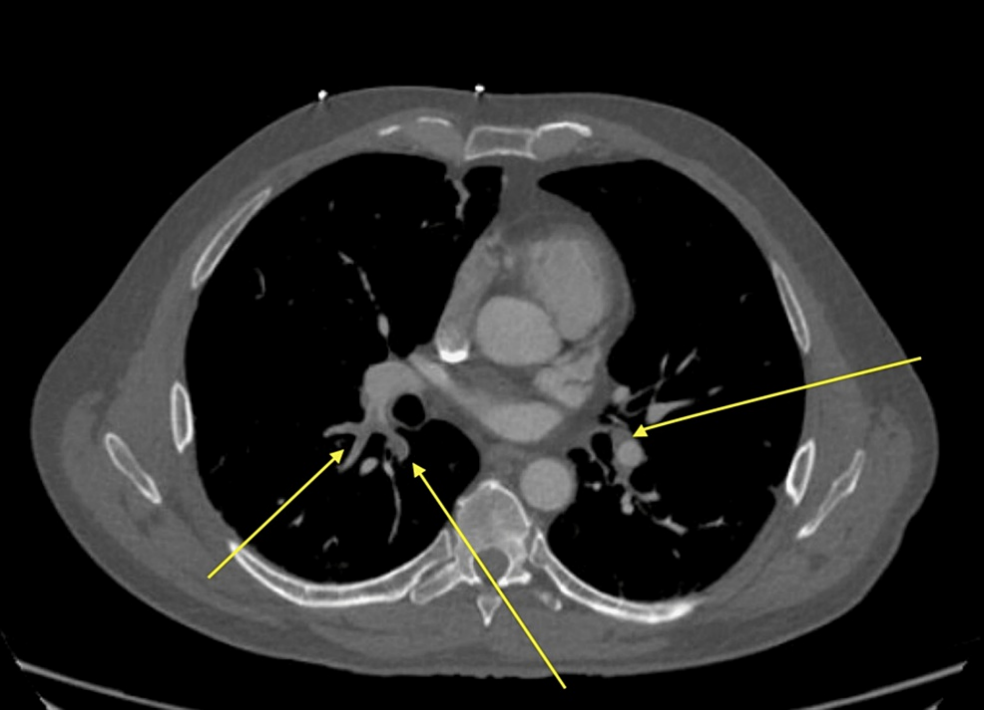
Day one, CT chest angiogram revealed pulmonary embolism within the segmental and sub-segmental branches of the right lung and sub-segmental branches of the left upper and right middle lobes (yellow arrows)

On a computed tomographic (CT) angiogram, the patient was found to have a pulmonary embolism, Figure 1, and was started on a heparin drip. Subsequent monitoring of partial thromboplastin time (PTT) remained subtherapeutic, and the patient was switched to Eliquis. The patient was closely monitored with a daily complete blood count (CBC). He then received hydralazine, labetalol, and amlodipine for uncontrolled HTN. Surgery service was consulted to evaluate RLE swelling with CT RLE, suggesting muscle contusion vs. soft tissue infection vs. hematoma.

The patient remained hemodynamically stable and was transferred to inpatient medicine service for management. He was started on IV Vancomycin and Ceftriaxone for fever with an unknown source of infection. The patient's condition gradually improved, and he became stable for discharge. The patient was discharged to home on HD day 13. See Table 1 for patient hospital course laboratory values.

**Table 1:** Laboratory values Creatinine kinase - CK U/L; blood urea nitrogen - BUN mg/dL; creatinine - Cr mg/dL; potassium - K+ mmol/L; bicarbonate - HCO3- mmol/L; phosphate - PO3- mg/dL; aspartate aminotransferase/ alanine aminotransferase - AST/ALT U/L

Labs	Day 1	Day 2	Day 3	Day 4	Day 5	Day 6	Day 7	Day 8	Day 9	Day 10	Day 11	Day 12	Day 13	Reference range
CPK	108,569	81,909	39,274	24,814	13,046	9,373	5,331	2,530	1,444	970	464	301	205	34-145 U/L
BUN	37	45	40	35	50	56	66	74	69	71	74	75	71	9-23 md/dL
SCr	3.4	4.3	4.8	5.5	7.2	7.7	7.9	7.8	6.6	5.8	5.0	4.7	4.1	0.6-1.0 mg/dL
K^+^	5.3	4.7	4.2	4.0	4.2	4.3	5.2	5.8	5.2	4.4	4.4	4.9	5.0	3.5-5.1
HCO_3_^-^	19	20	21	22	21	20	22	20	19	19	22	21	21	20-31 mmol/L
PO3_3_^-^	7.9	6.6	5.0	5.0	6.6	6.0	6.4	5.8	6.0	6.3	6.1	5.6	5.9	2.4-5.1 mg/dL
AST	2170	1604	1084	714	452	412	262	159	125	113	92	80	69	13-40 U/L
ALT	536	493	411	310	261	255	223	148	109	90	61	46	36	7-40 U/L

## Discussion

Antihistamines, such as doxylamine, represent standard components of over-the-counter sleep-inducing agents [11]. The easy availability of these substances increases the potential for both intentional overdose by adults and inadvertent ingestion by children [12]. As a result, rhabdomyolysis due to doxylamine overdose presents more frequently [13]. Clinicians must recognize the complications of rhabdomyolysis in patients who ingest doxylamine succinate and other over-the-counter antihistamines [12]. Drowsiness, seizure, sinus tachycardia, hypotension, and rhabdomyolysis represent clinical manifestations of acute H1 antihistamine intoxication [9, 12, 14]. Furthermore, doxylamine-overdose warrants early therapy as drug concentrations peak within two to three hours following ingestion [14]. Prompt intervention and careful assessment of renal function, urinary output, and serum creatine kinase (CK) levels may represent the difference between an uncomplicated course and ARF [12].

A past study explained drug-induced rhabdomyolysis via two mechanisms: direct drug injury to the striated muscle and local muscle compression in seizure, coma, and metabolic abnormality [5]. Another study suggested that antihistamines cause a direct toxic effect on striated muscle by injuring the sarcolemma leading to an influx of sodium. High intracellular sodium concentration activates the Na/K ATPase, ultimately depleting cellular ATP. In addition, increased intracellular sodium also causes higher levels of intracellular calcium, activating various proteolytic enzymes [15]. This combined depletion of ATP and activated intracellular enzymes lead to myocyte injury, rhabdomyolysis, and release of muscle contents such as CK and myoglobin, as seen in our case presentation [15].

Although the mechanism remains undefined, studies show that antihistamine overdose causes direct myocyte damage [13, 14]. Following skeletal muscle insult, circulating myoglobin causes oxidative injury to the kidney by forming F2-isoprostanes, leading to renal vasoconstriction. Myoglobin then induces renal failure via tubular obstruction by resultant myoglobin casts and lipid peroxidation-mediated tubular injury [16]. We hypothesize a similar process occurred in our patient. According to the literature, rhabdomyolysis in the context of doxylamine overdose is attributed to various factors, such as the quantity of doxylamine consumed, extended seizure episodes, co-ingestion of other drugs, or muscle compression leading to ischemic necrosis. These factors can result in a significant increase in CK levels [9, 12, 17]. We utilized this knowledge to guide the treatment process for our case.

Based on a recent multivariate regression analysis by Jo et al. [13], the quantity of doxylamine ingested was the sole reliable predictor for developing rhabdomyolysis following a doxylamine overdose. Specifically, rhabdomyolysis occurred significantly more frequently in patients who consumed doses exceeding 20 mg/kg compared to those taking lower doses. In addition, the study found an association between the amount of doxylamine ingested, the initial Cr level, and the initial arterial pH with rhabdomyolysis. However, this study did not find a statistically significant correlation between the ingested amount of the drug and peak levels of CK. 

Systemic toxicities are expected depending on plasma concentrations of doxylamine, with a likely positive correlation between muscle mass and CK level [14]. Therefore, clinicians should work up all patients with a doxylamine overdose and begin treatment for rhabdomyolysis to prevent AKI [13, 14]. Maintaining an aggressive volume expansion strategy using intravenous fluids is pivotal for preventing rhabdomyolysis-associated nephrotoxicity and acute renal failure caused by myoglobin. Scharman et al. [18] recommended the immediate initiation of fluid administration within the initial six hours following muscle injury. The suggested fluid rate should maintain a urine output of 300 mL/h or higher during the first 24 hours to mitigate the risk of ARF in patients with rhabdomyolysis. Furthermore, the use of intravenous sodium bicarbonate to correct systemic acidosis, if present, and the administration of mannitol to sustain the desired urine output, if needed. 

One study reported that Lactated Ringer's solution proved more valuable than normal saline in treating rhabdomyolysis secondary to doxylamine intoxication [19]. Kim et al. [14] reported that early alkalinization produces better outcomes, lowers the occurrence of rhabdomyolysis, and reduces the total hospital admission length in high-dose doxylamine overdose. Urine alkalinization increases the elimination of many compounds, including chlorpropamide, methotrexate, phenobarbital, salicylate, and other weak acid metabolites. Ionization of a weak acid increases in alkaline environments leading to increased excretion via the kidney [20]. Moreover, clinicians hypothesized that alkalinization of urine prevents renal vasoconstriction resulting in enhanced excretion of the toxic metabolites of doxylamine and myoglobin via renal tubules, thereby reducing peak serum concentration time and preventing direct renal tissue damage [14]. Subsequent studies should include the measurement of muscle mass and must investigate the correlation between urine alkalinization and doxylamine serum level [14]. 

## Conclusions

Doxylamine associated with acute kidney injury from rhabdomyolysis can result in poor prognosis, necessitating emergent hemodialysis, and critical care management, as seen in our patient. The accessibility of this drug, alongside its potential for abuse, warrants discussion among healthcare providers. Additionally, the adverse outcomes associated with doxylamine overdose demand that clinicians act immediately in treating patients with suspected intoxication. Rapid intervention may prevent the progression of renal failure, ultimately reducing the risk of developing chronic kidney disease and requiring long-term dialysis.
